# Kilohertz-frequency interferential current induces hypoalgesic effects more comfortably than TENS

**DOI:** 10.1038/s41598-023-35489-7

**Published:** 2023-05-27

**Authors:** Dahoon Park, Yushin Kim

**Affiliations:** Department of Sports Rehabilitation, Cheongju University, Cheongju, South Korea

**Keywords:** Electrical and electronic engineering, Pain, Peripheral nervous system

## Abstract

Recent research on transcutaneous electrical stimulation has shown that inhibiting nerve conduction with a kilohertz frequency is both effective and safe. This study primarily aims to demonstrate the hypoalgesic effect on the tibial nerve using transcutaneous interferential-current nerve inhibition (TINI), which injects the kilohertz frequency produced by the interferential currents. Additionally, the secondary objective was to compare the analgesic effect and comfort of TINI and transcutaneous electrical nerve stimulation (TENS). Thirty-one healthy adults participated in this cross-over repeated measures study. The washout period was set to 24 h or more. Stimulus intensity was set just below the pain threshold level. TINI and TENS were applied for 20 min each. The ankle passive dorsiflexion range of motion, pressure pain threshold (PPT), and tactile threshold were measured at the baseline, pre-test, test (immediately before ceasing intervention), and post-test (30 min after ceasing intervention) sessions. After the interventions, the participants evaluated the level of discomfort for TINI and TENS on a 10 cm visual analog scale (VAS). As the results, PPT significantly increased compared to baseline in test and posttest sessions of TINI, but not in those of TENS. Also, participants reported that TENS was 36% more discomfort than TINI. The hypoalgesic effect was not significantly different between TINI and TENS. In conclusion, we found that TINI inhibited mechanical pain sensitivity and that the inhibitory effect persisted long after electrical stimulation ceased. Our study also shows that TINI provides the hypoalgesic effect more comfortably than TENS.

## Introduction

47% of the adult population had musculoskeletal pain lasting more than a week in the past month^1^. To reduce musculoskeletal pain, clinicians have commonly use non-invasive electrical stimulation techniques^2,3^. For instance, based on the gate control theory, low-frequency electrical stimulation, known as transcutaneous electrical nerve stimulation (TENS), has been widely applied to reduce musculoskeletal pain by intentionally stimulating superficial skin mechanoreceptors and nociceptors^4^. However, since the existing literature doubts that TENS might be ineffective or inconclusive for musculoskeletal pain^5,6^, continuous technical development is needed.

The frequency range of TENS, which is well-known in clinical practice, is 1 to 100 Hz. However, some clinical researchers have suggested using frequencies higher than 1 kHz to reduce pain^3,7^. Specifically, electrical stimulation with a kilohertz-frequency alternating current has been introduced for a completely different mechanism from the conventional TENS^8^. Previous animal studies have demonstrated that kilohertz-frequency electrical stimulation can block peripheral nerve conduction directly^9,10^. Also, a clinical study demonstrated the efficacy and safety of kilohertz-frequency electrical stimulation for relieving postamputation pain^7^. Based on this essential evidence, subsequent studies have developed a non-invasive nerve inhibition technique using surface electrodes to compensate for the limitations of surgical approaches that require the direct insertion of electrodes into peripheral nerves^11–13^. Since this non-invasive method has the advantage of having high universality compared to the surgical technique, continuous research on this will be required.

When applying electrical nerve stimulation by the non-invasive method, the distance between the surface electrode and the nerve is the major constraint. To overcome this limitation, an interferential current has been developed. The interferential current therapy uses the mechanism that the skin resistance decreases as the electrical stimulation frequency increases^14^, allowing the current to penetrate deeper tissue. The main characteristic of the interferential current therapy is that when two different carrier frequencies are applied to the human body, a new beat current, corresponding to the difference in two carrier frequencies, is formed in a deep tissue area between the surface electrodes. For example, in clinical practice, carrier frequencies of 4000 Hz and 4050 Hz are simultaneously applied to the pain area to deliver a 50 Hz interference current to a deeper area. In current literature, the interferential current has been used for the pain control effect as an alternative to TENS using 100 Hz or less^15–18^. However, studies on the interferential current that produces kilohertz beat frequency for pain control have not been conducted yet.

Therefore, we propose a new technique using the beat current of kilohertz frequency, namely transcutaneous interferential-current nerve inhibition (TINI). Given the mechanism of interferential current therapy, this technique can alleviate the limitation of penetrating distance when electrical currents are delivered into the body through surface electrodes. In practice, the higher the available frequency, the less the effect of skin impedance^19^. That is since TINI uses a high carrier frequency, it is less affected by skin resistance. Hence, we hypothesize that TINI stimulates deep tissues such as the peripheral nerve but less cutaneous nerve.

This study aims to demonstrate the feasibility of TINI as a new pain control technique. To identify the feasibility, the hypoalgesic effects of TINI were demonstrated using mechanical somatosensory tests. The main hypothesis of the study was that TINI alters pressure pain threshold (PPT), tactile threshold (TT), and join range of motion (ROM) compared with baseline. Moreover, we compared the hypoalgesic effects of TINI with those of TENS, the most popular method in non-invasive pain control, such that as the second hypothesis, we expected that TINI is superior to conventional TENS on hypoalgesia. For an experiment to verify the hypothesis, we repeatedly applied TENS and TINI to the lower extremities of healthy participants by using a crossover design and compared the hypoalgesic effects of the two techniques. In addition, the practicality of the technique was evaluated by examining the level of subjective discomfort from electrical stimulation.

## Methods

This study included 31 healthy adults. Table 1 displays the demographic characteristics of the participants. Participants were excluded if they reported acute or chronic pain, musculoskeletal discomfort, diabetes, high blood pressure, pregnancy, an autoimmune disease, a history of surgery, neurological disorders, skin problems, or if they were using any type of pain medication. Additionally, those with a pacemaker or an intrinsic stimulator were also excluded. Before enrollment, a written agreement was acquired from all participants. And this study was conducted by the principles of the Declaration of Helsinki. The Institutional Review Board authorized the procedure for the experiment.

**Table 1:** Demographic characteristics.

Variable	N = 31 (Mean ± SD)
Women/Men (n/n)	11/20
Age (year)	23.26 ± 4.83
Height (cm)	173.13 ± 8.03
Weight (kg)	70.12 ± 16.72

SD: Standard deviation.

### Procedure

**Figure 1:**
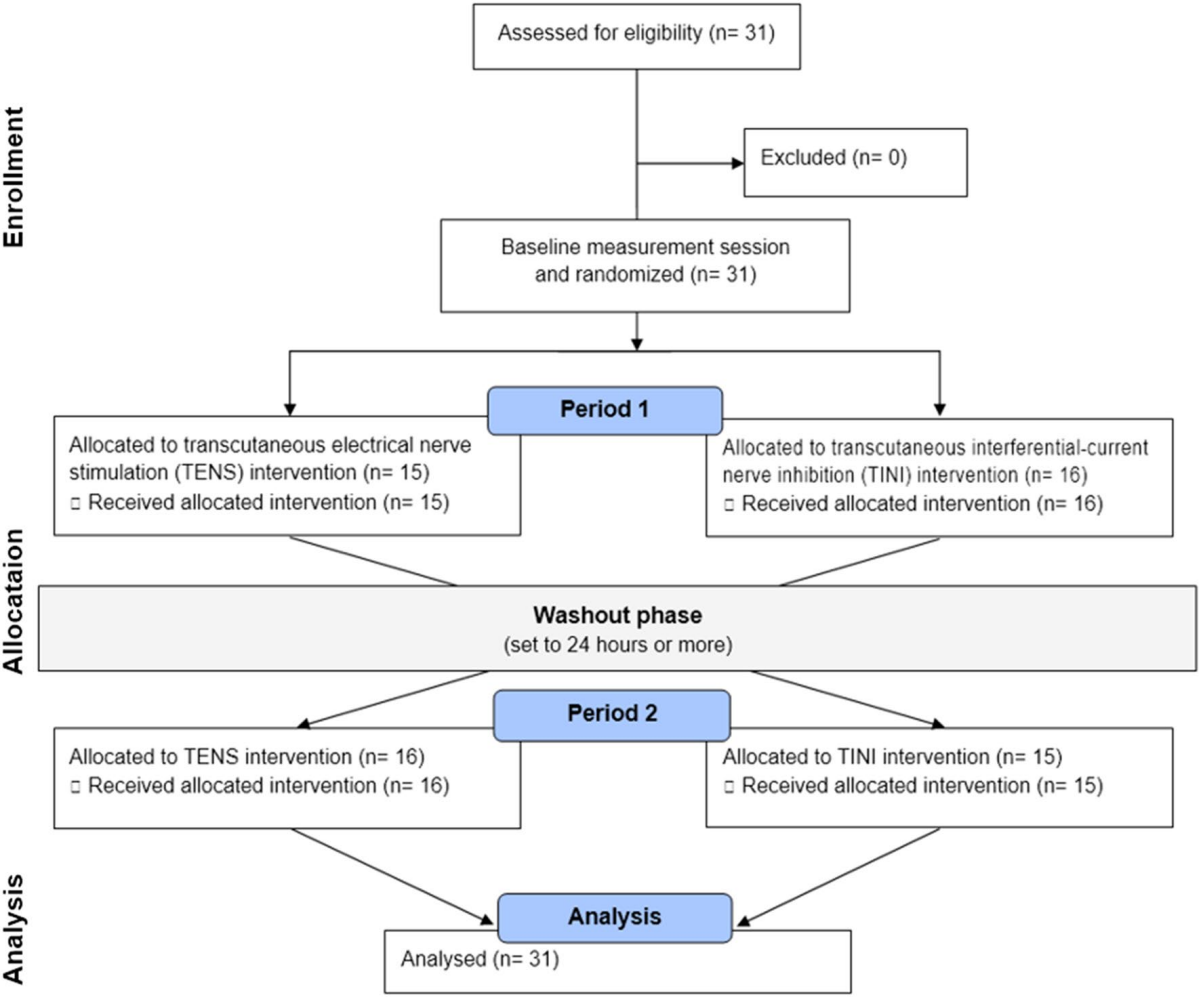
The flow diagram of participants.

In this study, TENS and TINI were applied to all subjects as a cross-over repeated measures design. All participants initially participated in a baseline measurement session. The next day we determined the order of application of two electrical stimuli per participant using simple randomization. Specifically, if an odd number was found in the random number generator, TINI was applied first, and TENS was applied after the washout period. If an even number is obtained, the reverse order was performed. The washout period was set to 24 h or more. Figure 1 displays the flow chart of the experimental procedure.

### Electrical stimulation

In the study, electrical stimulation aimed to stimulate the plantar area of the participants’ left foot, the non-dominant leg. TINI, which targets peripheral nerve stimulation according to the principle of technology application, focuses on stimulating the tibial nerve that dominates the plantar area. On the other hand, TENS focuses on stimulating the cutaneous nerves in the target area based on the gate control theory.

**Figure 2:**
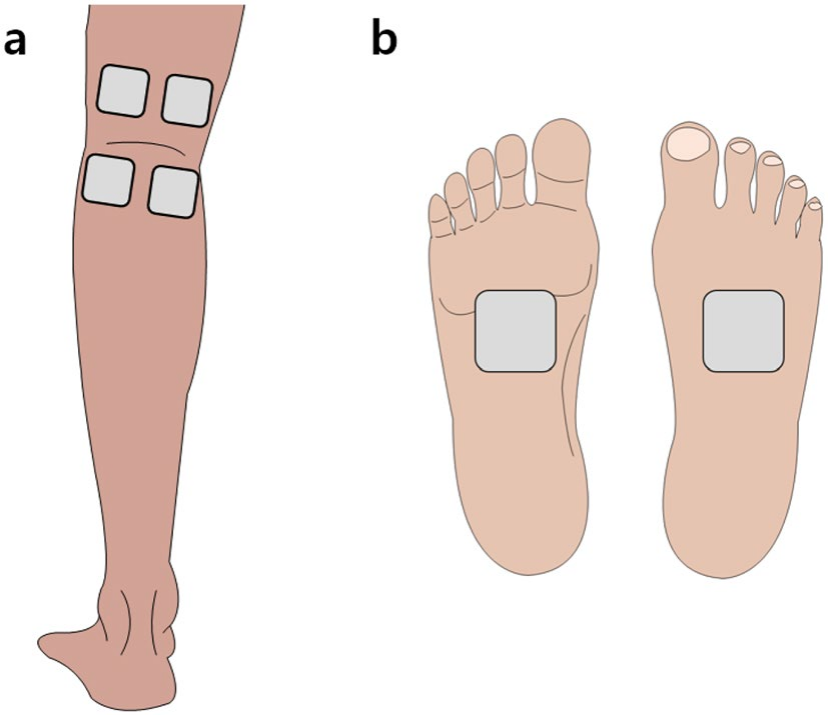
The electrode placement of transcutaneous interferential-current nerve inhibition (TINI) (**a**) and transcutaneous electrical nerve stimulation (TENS) (**b**). The gray squares represent the electrodes.

**Figure 3:**
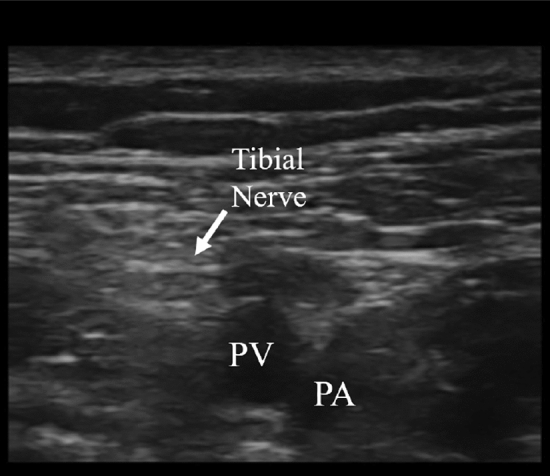
Ultrasound image for finding the tibial nerve. PA: Peroneal artery, PV: Peroneal vein.

TINI was administered through a surface electrode (40 × 40 mm, square shape, BioProtec, Wonju, Korea) using interferential current equipment (T-1000, B&C Healthcare, Seoul, Korea). An oscilloscope (TDS2012C, Tektronix, Beaverton, OR, USA) was used to confirm that each channel of the device produces charge-balanced, biphasic, and unmodulated sine wave. In the study, TINI was given at frequencies of 20 and 21 kHz, so the interferential current was 1 kHz. Four electrodes were located in the center of a popliteal region to deliver the interferential current into the tibial nerve (Fig. 2a). Before the attachment of electrodes, we identified the location of the tibial nerve using ultrasound images (Fig. 3). Each electrode was placed 1 cm apart centered the location of the tibial nerve. The upper two electrodes were connected to the first channel at 21 kHz, and the lower two were connected to the second channel at 20 kHz.

As a comparative variable, TENS was performed using the same surface electrode. Popular TENS equipment (InTENSity Select Combo II, METICULOUS MEDICAL, San Jose, USA) was used. We identified the TENS device produces quadrature waves and 80–120 Hz. the pulse duration was modulated from 100 to 120 us with 10 s. Two electrodes were placed on the dorsal and plantar surfaces (Fig. 2b).

Stimulus intensity of TINI and TENS was set just below the pain threshold level, i.e., the maximum intensity that produced brief tingling but no pain^20^. When evaluating the pain threshold, we also examined whether participants felt electrical stimulation at their feet. Moreover, if electrical stimulation elicited tetanus contractions in the calf or foot muscles, we decreased the stimulus intensity until the tetanus contractions subsided. After the electrode position and stimulus intensity were determined, TINI and TENS were applied for 20 min, respectively.

### Measurements

In this study, the pressure pain threshold (PPT), tactile threshold (TT), and passive range of motion (ROM) of dorsiflexion of the ankle were tested to evaluate the effect of electrical stimulation. Measurements were performed at baseline and pre-test, test, and post-test of the stimulation. All participants performed baseline measurements without knowing which intervention they would participate in first. The pretest was conducted immediately before the application of electrical stimulation. Then, the test was performed 20 min after beginning electrical stimulation, i.e., immediately before ceasing stimulation. The posttest was carried out 30 min after the electrical stimulation was finished. As a result, seven measurements were repeated for each participant since TINI and TENS were applied through the crossover design.

PPT was measured using a pressure algometer (FPK 20; EFFEGI, Italy) to determine the mechanical pain threshold. The dial gauge’s limits were between 0 and 10 kg/cm^2^. Mechanical pressure was administered to the calf, heel, 1st-toe, and 5th-toe, referring to the measurement method for a patient with rheumatoid arthritis^21^. The pressure increased at a rate of 1 kg/s until the subject reported their first pain experience, and the pressure applied at that moment was recorded. To measure TT, we used a set of 20 Semmes–Weinstein monofilaments (Touch Test Sensory Evaluators, North Coast Medical, Gilroy, CA, USA). The measurements began with the monofilament with the smallest diameter, the ascent technique of the threshold test^20^. The monofilament was held in contact with the skin until it bent, then detached after 1 s. Participants were instructed to close their eyes and indicate if they could perceive skin pressure from a monofilament. The manufacturer-specified milligram force and force values were shown on a logarithmic scale^21^. Ankle ROM was assessed using a passive continuous machine (CPM) and an electronic inclinometer (Nippon Medical and Chemical Instruments, Japan). The inclinometer was placed on a foot plate of the CPM. The measurement was initiated at the vertical position and stopped when the participant felt stretching pain. All measurements were repeated three times for a session, and the mean of the measured values was used for the analysis.

The visual analog scale (VAS) was also performed to evaluate the discomfort level of electrical stimulation quantitatively. Participants marked their discomfort between a straight line of 10 cm, with points 0 (no discomfort at all) and 10 (pain beyond discomfort) at either end. VAS was measured at the end of the experiment. During the experiment, the researcher, who has clinical experience, monitored for several potential adverse effects of electrical stimulation, including burns, redness, swelling, muscle soreness, and muscle twitching or spasm^22–25^.

### Statistical analysis

To test the main hypothesis, we compared PPT, TT, and ROM between the baseline and intervention sessions (pre-test, test, and post-test of TINI and TENS, respectively) using one-way repeated measures analysis of variance (RMANOVA). Effect sizes were also calculated using Cohen’s d and will be reported alongside the results of the statistical tests^26^. For the second hypothesis, we compared PPT, TT, and ROM between TINI and TENS conditions using two-way RMANOVA (three-time factors: pre-test, test, and post-test; two intervention factors: TINI and TENS). If significant differences were detected after analysis, post hoc contrast tests were used to examine the statistically significant differences in measurement values. To assess the test–retest reliability of the measurements, we conducted intraclass correlation coefficients (ICC) using a 2-way mixed-effects model (model 3,3)^27^. The paired t-test compared the VAS scores for discomfort between TENS and TINI. Statistical analysis was performed using SPSS version 26.0 (IBM Corporation, Armonk, NY, USA), with a significance set at p < 0.05.

### Ethical approval

Approval was granted by the Institutional Review Board of Cheongju University, Cheongju, Korea (1041107-202206-HR-021-01).

### Consent to participate

Prior to participation, all individuals included in the study provided written informed consent for their involvement.

## Results

**Figure 4:**
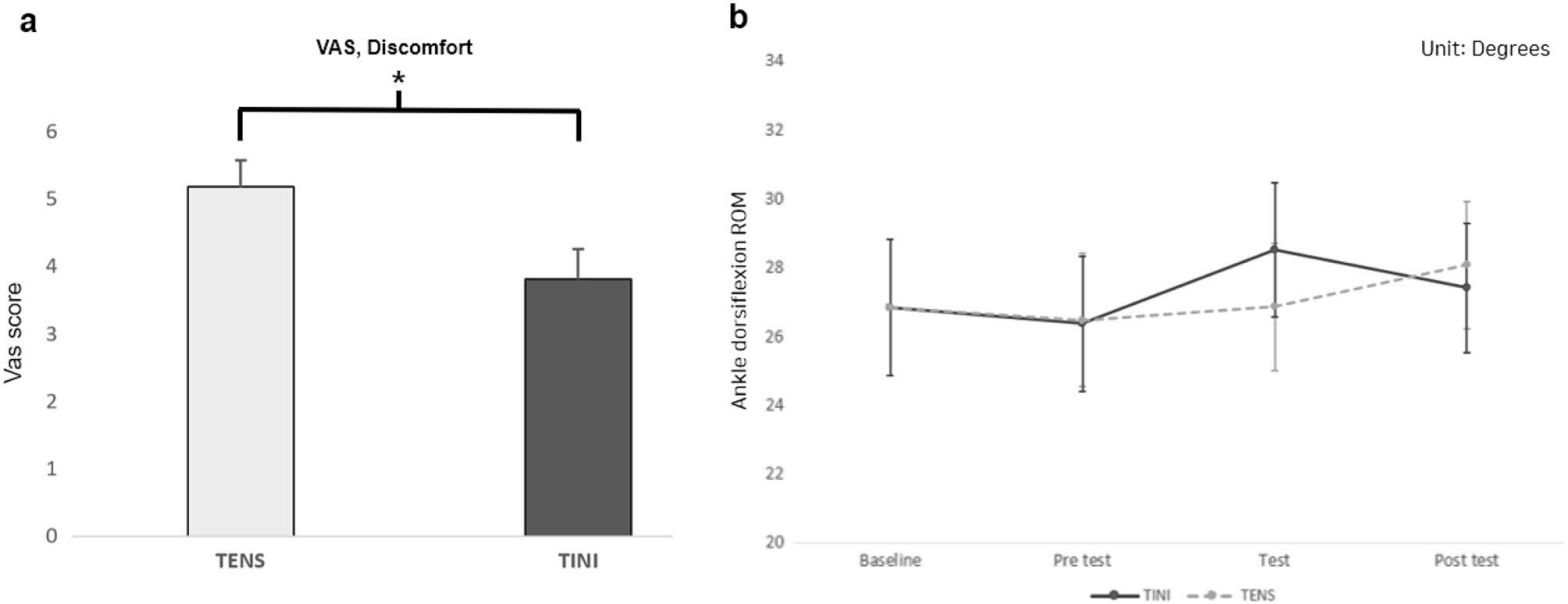
The visual analog scale (VAS) score for the discomfort of electrical stimulation (**a**). And the ankle dorsiflexion range of motion (ROM) at each condition (**b**). The data are shown as group means ± standard error. *p < 0.05. TENS: Transcutaneous electrical nerve stimulation group, TINI: Transcutaneous interferential-current nerve inhibition group.

The VAS for quantifying the discomfort of electrical stimulation was significantly different between TENS (5.18 ± 0.39) and TINI (3.81 ± 0.45) (Fig. 4a). Our participants reported that TENS was 36% more discomfort than TINI (t = 2.62, p = 0.014).

**Figure 5:**
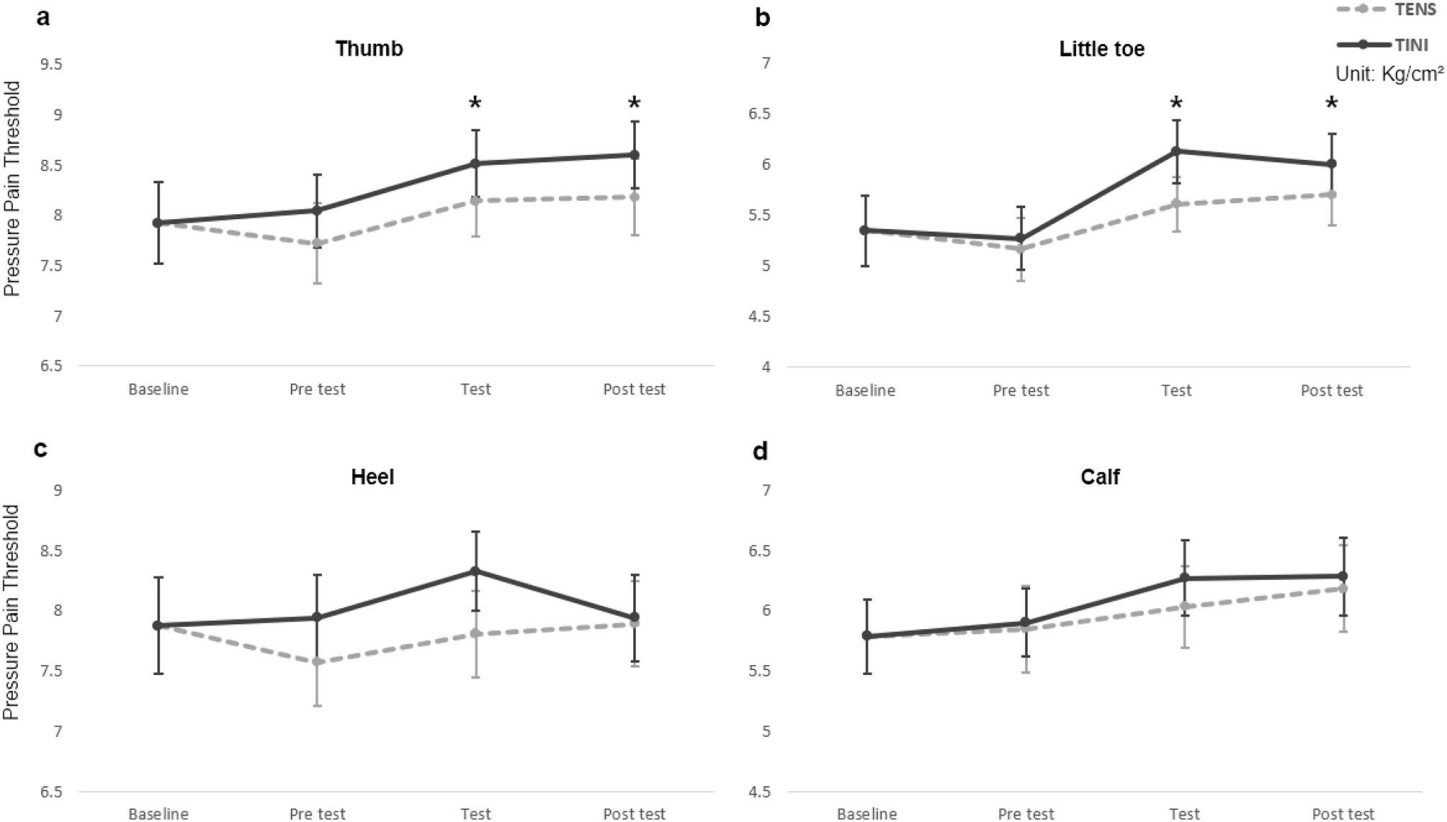
The pressure pain threshold (PPT) at each condition (**a**: thumb, **b**: little toe, **c**: heel, **d**: calf). The data are shown as group means ± standard error. *p < 0.05. TENS: Transcutaneous electrical nerve stimulation group, TINI: Transcutaneous interferential-current nerve inhibition group.

**Figure 6:**
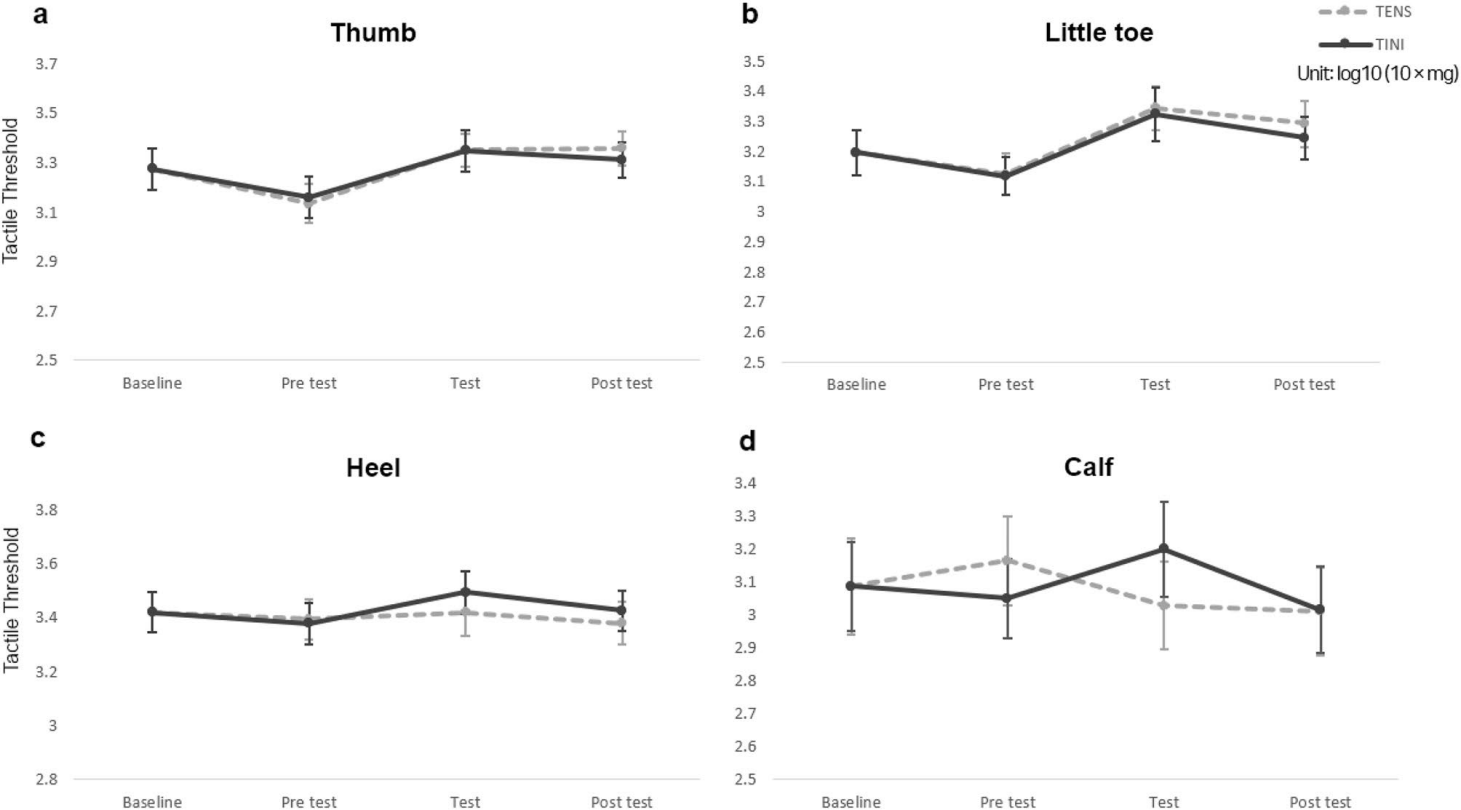
The tactile threshold (TT) at each condition (**a**: thumb, **b**: little toe, **c**: heel, **d**: calf). The data are shown as group means ± standard error. *p < 0.05. TENS: Transcutaneous electrical nerve stimulation group, TINI: Transcutaneous interferential-current nerve inhibition group.

Regarding the mechanical pain threshold, we confirmed that TINI induced significant changes. Compared to the baseline, TINI significantly increased PPT measurements (Fig. 5). One-way RMANOVA showed significant changes in PPT for both the thumb (F = 2.851, p = 0.030) and the little toe (F = 3.881, p = 0.007). In the post hoc analysis, the PPT of the thumb significantly increased by 7.45% (d =  − 0.284, p = 0.029) in the test session (8.51 ± 0.33) and by 8.57% (d =  − 0.326, p = 0.014) in the post-test (8.60 ± 0.34) of TINI compared to the baseline (7.92 ± 0.41). Similarly, the PPT of the little toe significantly increased by 14.65% (d =  − 0.424, p = 0.025) in the test session (6.13 ± 0.31) and by 12.35% (d =  − 0.357, p = 0.032) in the post-test (6.00 ± 0.30) of TINI compared to the baseline (5.34 ± 0.35). PPT changes following the TENS application did not show statistically significant differences compared to the baseline. In TT and ROM, all measured values showed no statistically significant differences compared to the baseline. On average, all measurements for both TT (Fig. 6) and ROM (Fig. 4b) demonstrated noticeable changes in the TINI or TENS compared to the baseline, but these changes were not statistically significant.

No significant main effect of treatment (TINI vs TENS) was found for using two-way RMANOVA. We observed significant main effects of time on PPT for the thumb (F = 7.443, p = 0.001), little toe (F = 12.269, p < 0.001), and calf (F = 6.548, p = 0.002), as well as in TT for the thumb (F = 15.255, p < 0.001) and little toe (F = 11.901, p < 0.001). However, no significant main effects of time were found in PPT for the heel and TT for the heel and calf. Additionally, no interactions were identified.

The range of ICC values for three times of repeated measurements in the study was 0.800 to 0.989. The electrical stimulation intensity of TENS was 60.42 ± 3.25 mA, TINI was 123.03 ± 9.35 mA (first channel), and 111.74 ± 8.71 mA (second channel). No adverse effects were observed in the participants.

## Discussion

This study aimed to determine the effectiveness of TINI in hypoalgesia and to compare its hypoalgesic effects with TENS, a non-invasive method widely used to suppress musculoskeletal pain^28^. Our results demonstrated a significant hypoalgesic effect of TINI, an interferential current composed of kilohertz-level beat currents. Furthermore, we found evidence that TINI provides a more comfortable experience than conventional TENS. However, we did not find any evidence to suggest that TINI's analgesic effect is superior to that of TENS.

The results of PPT showed that TINI decreased mechanical pain sensitivity, and its suppressing effect remained 30 min after the cessation of electrical stimulation. In a similar previous study comparing PPT between TENS and interferential currents (IFC)^18^, the average change rate was 11.15% (8.16% to 17.65%) immediately after the intervention. The results of our study showed a 7.45% increase in the thumb and a 14.65% increase in the little toe during the test session, and an 8.57% increase in the thumb and a 12.35% increase in the little toe during the post-test session, are consistent with the findings of a previous study^18^.

The less discomforting pain-relieving effect of TINI is likely attributable to a different neurological mechanism compared to that of TENS. Pain suppression of TENS is based on gate control theory^29,30^. According to the gate control theory, the activity of A-beta fibers that transmit the sense of touch excites substantia gelatinosa neurons, and these intermediate neurons reduce pain through synaptic transmission inhibition^29,30^. As a result, it is favorable for the patient to perceive electrical stimulation to alleviate pain. However, as a drawback of TENS, the perception of electrical stimulation can be very unpleasant for some patients. In addition to pain reduction by the gate control theory, TINI was presumed to inhibit peripheral nerve conduction. Specifically, kilohertz electrical stimulation of TINI inhibits peripheral nerve conduction by inactivation of sodium channels due to extensive depolarization^31,32^ or continuous activation of potassium channels to prevent action potential propagation^33,34^. Moreover, high-frequency neural fatigue, in which neurotransmitters are depleted, could be one of the pain suppression mechanisms of TINI^3^. This peripheral nerve conduction inhibitory action is presumed to be the leading cause of TINI, reducing pain with more comfortable electrical stimulation. Lastly, less stimulation of the cutaneous nerve in TINI, due to low skin impedance by high frequency^19^, might reduce pain more comfortably.

Due to the differences in the neurological mechanisms of TENS and TINI, the application methods and electrode placement of the two electrical stimulations were different. TENS has been applied near the site of pain^35–37^, while TINI focuses on inhibiting nerve conduction^11,38,39^. In accordance with this concept, TINI has been applied not to the location of pain but to the nerve that innervates the area of pain. Thus, in this study, TINI was applied to the popliteal area by targeting the tibial nerve, which innervates the sole. As a result, the hypoalgesic effect was observed in the thumb and little toe innervated by the tibial nerve but not in the calf regions innervated by the saphenous or sural nerve. We understand that the different electrode placements might influence the analgesic effect comparison between TINI and TENS. However, the electrode positions were chosen based on the distinct mechanisms of each stimulation method. We expect this TINI method to be an alternative that can be applied to patients with difficulty attaching electrodes directly to the painful area.

This study showed no significant change in ankle ROM and TT during and after electrical stimulation. In both parameters, the average value increased from the baseline but was not statistically significant. Although we expected increased ROM due to neurological hypoalgesia, short-term stretching may limit the increasing length of non-contractile tissues such as fascia and joint capsules. In the PPT, unlike the toes, there was no difference in the analgesic effect of electrical stimulation, i.e., TINI, on the heel compared to the baseline. Considering that the heel area, where the PPT was measured, is innervated by the medial calcaneal nerves, we assume that the corresponding nerve stimulation was insufficient. Therefore, it is necessary to develop advancements in stimulation location and parameter setting to improve effective stimulation technology in the future. In addition, the small number of subjects may have adversely affected the statistical significance test. Through future studies, it is necessary to observe changes in ROM and TT of TINI in more participants over a long period.

In our study, we assessed various potential adverse effects, including burns, redness, swelling, muscle soreness, and muscle twitching or spasm, as reported in previous studies^22–25^. Transcutaneous electrical stimulation has been reported to be generally safe, with a low risk of infection or tissue damage as it does not involve invasive procedures or foreign substances injected into the body^40^. We observed no adverse effects caused by the electrical stimulation in the participants, as mentioned above. Therefore, we expect that applied TINI below the pain threshold level to be safe.

The limitation of the study is that our participants were not patients, so different results may appear in patients having neurologic, musculoskeletal, or rheumatic pain. Another limitation of our study is the absence of a sham control group. Including a sham control group in future research could help provide a more comprehensive view of the analgesic effects of both TINI and TENS. Although TENS demonstrated a low analgesic effect in this study, it cannot be concluded that it lacks therapeutic value. For instance, a higher analgesic effect is typically achieved in clinical practice by gradually increasing the stimulation intensity after a 5-min adaptation period^41^. It is anticipated that both TINI and TENS would yield stronger pain reduction effects if the stimulation intensity is progressively increased through an adaptation period. Based on our findings, which demonstrate a hypoalgesic effect with no adverse effects, we expect that follow-up studies targeting patients will be conducted.

## Conclusion

This study demonstrated that TINI reduced mechanical pain sensitivity, with the inhibitory effect persisting even after the cessation of electrical stimulation. Additionally, we found TINI to be more comfortable than traditional TENS for pain reduction. In this study, TINI exhibited potential as an alternative hypoalgesic agent for patients who have difficulty using TENS.

## Data Availability

The datasets for the current study are available and will be provided by the corresponding author Yushin Kim (kimy@cju.ac.kr) upon reasonable request.
